# Take a stand on understanding: electrophysiological evidence for stem access in German complex verbs

**DOI:** 10.3389/fnhum.2015.00062

**Published:** 2015-02-26

**Authors:** Eva Smolka, Matthias Gondan, Frank Rösler

**Affiliations:** ^1^Department of Linguistics, University of KonstanzKonstanz, Germany; ^2^Department of Psychology, University of CopenhagenCopenhagen, Denmark; ^3^Biological Psychology and Neuropsychology, University of HamburgHamburg, Germany

**Keywords:** event-related potentials, derivational morphology, morphological priming, semantic priming, form priming, stem access, complex verbs

## Abstract

The lexical representation of complex words in Indo-European languages is generally assumed to depend on semantic compositionality. This study investigated whether semantically compositional and noncompositional derivations are accessed via their constituent units or as whole words. In an overt visual priming experiment (300 ms stimulus onset asynchrony, SOA), event-related potentials (ERPs) were recorded for verbs (e.g., *ziehen*, “pull”) that were preceded by purely semantically related verbs (e.g., *zerren*, “drag”), by morphologically related and semantically compositional verbs (e.g., *zuziehen*, “pull together”), by morphologically related and semantically noncompositional verbs (e.g., *erziehen*, “educate”), by orthographically similar verbs (e.g., *zielen*, “aim”), or by unrelated verbs (e.g., *tarnen*, “mask”). Compared to the unrelated condition, which evoked an N400 effect with the largest amplitude at centro-parietal recording sites, the N400 was reduced in all other conditions. The rank order of N400 amplitudes turned out as follows: morphologically related and semantically compositional ≈ morphologically related and semantically noncompositional < purely semantically related < orthographically similar < unrelated. Surprisingly, morphologically related primes produced similar N400 modulations—irrespective of their semantic compositionality. The control conditions with orthographic similarity confirmed that these morphological effects were not the result of a simple form overlap between primes and targets. Our findings suggest that the lexical representation of German complex verbs refers to their base form, regardless of meaning compositionality. Theories of the lexical representation of German words need to incorporate this aspect of language processing in German.

## Introduction

One intriguing question in psycholinguistic and neurolinguistic research is how morphologically complex words like *understand* are represented in lexical memory, via their base {stand} or via the whole form? Traditional means to study the lexical memory of complex words have been (a) the use of overt priming conditions; and (b) the manipulation of semantic compositionality between morphologically related words. Overt priming conditions such as auditory or visual priming at long exposure durations (230–250 ms stimulus onset asynchrony (SOA) or longer) guarantee that the prime is consciously perceived. Under these conditions, semantic processing takes place and the meaning of the word can be retrieved. Overt priming can thus be used to tap into lexical memory.

The manipulation of semantic compositionality has been a further means to study lexical representations. For example, the meaning of the word *underdress* is semantically transparent (i.e., compositional), since it can be derived from the meaning of its morphemic constituents. The priming of a word like *dress* by *underdress* can thus be attributed to either morphological or semantic relatedness between the two words or both. By contrast, *stand* and *understand* are purely morphologically related, since the meaning of *understand* is semantically opaque and cannot be composed of the meaning of its parts. Any facilitation of the target *stand* by the word *understand* cannot be attributed to a meaning relation between the two words. Such facilitation would rather stress the morphological relatedness between the words, indicating that the two share some lexical representation in spite of their opaque meaning relation. In other words, the facilitation of *stand* by *understand* would indicate that *understand* is lexically represented via its base {stand}.

Behavioral findings in Indo-European languages such as English, French, and Dutch suggest that the lexical representation of complex words depends on semantic compositionality: Stems like *confess* were primed by semantically transparent derivations like *confessor*, but stems like *success* were not facilitated by morphologically related but semantically opaque derivations like *successor* (for cross-modal priming, see Marslen-Wilson et al., 1994; Longtin et al., 2003; for visual priming at long SOAs, see Feldman and Soltano, 1999; Rastle et al., 2000; Feldman et al., 2004). Similar to the stimuli used in the present study, also prefixed derivations like *distrust* primed their stems like *trust*, as well as other prefixed or suffixed derivations like *entrust* or *trustful*, though only if they were semantically transparent and not if they were semantically opaque (Marslen-Wilson et al., 1994; Feldman and Larabee, 2001; Meunier and Segui, 2002).

These findings were taken to indicate that overt priming triggers morphological decomposition as a high-level process, either following whole word access or constrained by semantic knowledge. Morphological models assume that semantically transparent words like *confessor* possess a lexical entry that corresponds to their base, such as {confess} and the suffix {or}, while semantically opaque words like *successor* must be represented in their full form (Rastle et al., 2000, 2004; Taft and Kougious, 2004; Diependaele et al., 2005; Meunier and Longtin, 2007; Marslen-Wilson et al., 2008; Taft and Nguyen-Hoan, 2010). By contrast, the distributed connectionist or *convergence of codes* view assumes that the above findings of morphological effects (whether or not priming occurs between morphologically related words) do not depend on explicit representations but rather on the degree of shared semantic and form similarity between the words (e.g., Plaut and Gonnerman, 2000; Gonnerman et al., 2007; Kielar and Joanisse, 2011). Indeed, under cross-modal priming conditions the priming effects of morphologically related (and hence form-related) prime-target pairs varied with the degree of their shared meaning (Gonnerman et al., 2007). Pairs with no or little shared meaning like *hardly-hard* showed no priming at all, those with moderate semantic similarity like *lately-late* showed medium effects, and those with high semantic similarity like *boldly-bold* showed the strongest priming effects. Most importantly, though, and regardless of whether morphological structure is assumed to be explicitly or implicitly represented, all of the above models assume that the semantic compositionality of a word determines its conscious processing and representation.

In contrast to overt priming, evidence for morphological priming without a semantic relation occurred in English or French, but they occurred only when the participants were unaware of the prime. Under masked priming conditions (and visual prime presentations below 50 ms SOA), bases were not only primed by morphologically related and semantically transparent words (e.g., *successful-success*) but also by semantically opaque derivations (e.g., *successor-success*), by pseudoderivations (e.g., *corner-corn*), and by nonwords that comprise a stem and an affix (e.g., *volter-volt). The priming of the latter two types has been taken to indicate that any morpheme-like ending (e.g., *-er*) induces a segmentation process without differentiating between real morphological derivations (e.g., *successful* and *successor*), pseudoderivations (e.g., *corner*) or nonwords (e.g., *volter). Such a differentiation occurs only under lexical processing (e.g., Rastle et al., 2000, 2004; Longtin et al., 2003; Diependaele et al., 2005; Marslen-Wilson et al., 2008; McCormick et al., 2009).

Integrating these data on prelexical and lexical processing, recent models of morphological processing (e.g., Rastle et al., 2000, 2004; Longtin et al., 2003) assume that the word recognition process occurs in two stages: In the early prelexical stage, complex words are decomposed on a purely orthographic basis, independent of true morphological or semantic compositionality. In the second, lexical stage, the morphemic (or morpheme-like) constituents are reappraised for semantic and syntactic information. As soon as semantic integration occurs, only semantic compositionality (but not the purely orthographically based segmentation process) affects the word recognition process.

However, since behavioral data (e.g., response times and errors) represent the endpoints of processing and decision making, the above theories of morpho-lexical processing leave some open questions concerning the processing and representation of morphologically complex words, in particular with respect to the time course of processing: Is there a sequential ordering of the different processing types? For example, does form processing occur prior to or simultaneously with morphological processing? Does semantic processing indeed occur alongside or after morphological processing? In this respect, electrophysiological evidence provides a useful means to answer these questions.

### Electrophysiological evidence for morphological effects

Event-related potentials (ERPs) derived from the electroence-phalogram before, during or after an event of interest have the advantage of revealing more directly differences of processes intervening between input and output than it is the case for behavior indices as reaction time and error rate. The latter measures are necessarily omnibus measures in which stimulus- and response-related effects are integrated. In contrast, an ERP effect as the N400 effect is primarily related to differences in processes which mediate between input and output and which are largely independent of stimulus bound processes (e.g., perceptual discriminability) or response bound processes (e.g., response frequency). Evidence accumulated over the last 30 years revealed that the N400 effect is a sensitive measure of the effort required to process a word, and the amplitude might be interpreted as reflecting the ease of memory access. The easier such access is, due to contextual, morphological or semantic priming, the smaller is the N400 effect (Kutas and Federmeier, 2011).

With respect to morphological processing, ERPs may reveal whether morphological priming effects resemble form processing that is reflected in early negativities or whether morphological effects ensue semantic memory access as reflected in N400 effects.

Similar to the behavioral studies, most ERP studies on morphological processing have applied repetition priming under masked or unmasked stimulus presentation. In the studies considered here, priming is concluded if the negative going ERP amplitude in the latency range of 250 ms (N250) or 400 ms (N400) is attenuated relative to an unrelated baseline condition (in which prime and target are neither semantically nor form-related), that is, to the most pronounced negativity. In other words, priming effects are concluded if the negativity in the N250 or N400 latency range is attenuated or reduced in the related condition relative to the unrelated condition (for a review, see Kutas and Federmeier, 2011).^1^ For a summary of morphological ERP effects induced by violation paradigms as compared to repetition priming, see Smolka et al. (2013). Table 1 of the present study summarizes the ERP findings of priming effects generated by real morphological derivations as compared to pseudoderivations or stem homographs, as compared to the effects of pure form or meaning relatedness.

**Table 1:** **Summary of ERP studies on complex word derivation using repetition priming**.

Study	Lang	PP	SOA	Task	Comparison	Morphological	Effect	Pseudocomplex/stem homograph	Effect	Form-related	Effect	Semantic	Effect
**Masked priming**
Holcomb and Grainger (2006)	E	v	#50#	SC	Unrelated vs. related	Identity (T) *mouth-TABLE* vs. *table-TABLE*	N250; P325; N400			Form-related *moath-TABLE vs. *teble-TABLE	Right ant. N250; N400
					T vs. F		N250 and N400: T > F
Morris et al. (2007)	E	v	#50#	LD	Unrelated vs. related	Transparent *shovel-HUNT* vs. *hunter-HUNT*	ant. N250* (200–300 ms); N400*	Pseudocomplex *actor-CORN* vs. *corner-CORN*	No effect*	Form-related *package-SCAN* vs. *scandal-SCAN*	No effect*
					T vs. P vs. F	*hunter-HUNT* vs. *corner-CORN* vs. *scandal-SCAN*	N250* and N400*: T > P > F **
Morris et al. (2008)	E	v	#50#	SC	Unrelated vs. related	Transparent *shovel-HUNT* vs. *hunter-HUNT*	N250 (200–300 ms)*	Pseudocomplex *actor-CORN* vs. *corner-CORN*	N250*	Form-related *package-SCAN* vs. *scandal-SCAN*	ant. N250*
					T vs. P vs. F	*hunter-HUNT* vs. *corner-CORN* vs. *scandal-SCAN*	N250*: T = P > F
			#100#		Unrelated vs. related		N250*; N400*		N250*; N400*		N250*; N400*
					T vs. P vs. F		N400*: T = P = F
Lavric et al. (2007)	E	v	#42	LD	Unrelated vs. related	Transparent *unrelated-HUNT* vs. *hunter-HUNT*	right post. N250 (220–260 ms); N400	Pseudocomplex *unrelated-CORN* vs. *corner-CORN*	ant. N250 (220–260 ms); N400	Form-related *unrelated-BROTH* vs. *brothel-BROTH*	Left ant. N250 (180–260 ms); N400
					T vs. P vs. F	*hunter-HUNT* vs. *corner-CORN* vs. *brothel-BROTH*	N400: T = P > F
Morris et al. (2011)	E	v	#50	LD	Unrelated vs. related	Transparent *painter-VOLT* vs. *voltage-VOLT*	N250 (225–325 ms), N400	Pseudocomplex *painter-VOLT* vs. *volter-VOLT	N250 (225–325 ms), N400	Form-related *painter-VOLT* vs. *voltire-VOLT	N250 (225–325 ms), N400
					T vs. P vs. F		N250 and N400: T = P = F
		v	#50	LD	Unrelated vs. related	Transparent *painter-VOLT* vs. *voltage-VOLT*	N250 (200–300 ms), N400	Pseudocomplex *paintity-VOLT vs. *volter-VOLT	N250 (200–300 ms), N400	Form-related *paintave-VOLT vs. *voltire-VOLT	N250 (200–300 ms), N400
					T vs. P vs. F		N250: T = P = F N400: T = P ≠ F
Morris et al. (2013)	E	v	#50	SC	Unrelated vs. related	Transparent *lendity-HUNTER vs. *huntity-HUNTER	P (150–200 ms), N250 (200–300 ms), N400	Pseudocomplex *towity-CORNER vs. *cornity-CORNER	N250, N400	Form-related *wallity-SCANDAL vs. *scanity-SCANDAL	N250, N400
					T vs. P vs. F		N250: T = P = F N400: T = P = F
**Overt priming**
Lavric et al. (2011)	E	v	226	LD	Unrelated vs. related	Transparent *unrelated-HUNT* vs. *hunter-HUNT*	N400 (320–480 ms)	Pseudocomplex *unrelated-CORN* vs. *corner-CORN*	N400 (300–430 ms)	Form-related *unrelated-BROTH* vs. *brothel-BROTH*	N400 (390–420 ms)
					T vs. P vs. F		N400: T > P > F
Barber et al. (2002)	S	v	250	LD	Unrelated vs. related	Inflection (T) *cera-LOCO* vs. *loca-LOCO*	N400	SHG *pera-RATO* vs. *rata-RATO*	N400; late N
					T vs. SHG	*loca-LOCO* vs. *rata-RATO*	N400: T > SHG
Domínguez et al. (2004)	S	v	300	LD	Unrelated vs. related	Inflection (T) *suma-PELO* vs. *bobo-BOBA*	P (250–350 ms); N400	SHG *suma-PELO* vs. *rata-RATO*	P (250–350 ms); N400, late N	Form-related *suma-PELO* vs. *toro-TONO*	No effect	Synonyms *suma-PELO* vs. *caldo-SOPA*	P (250–350 ms); N400
					T vs. SHG			*bobo-BOBA* vs. *rata-RATO*	P (250–350 ms): T = SHG N400: T > SHG
					T vs. F					*bobo-BOBA* vs. *toro-TONO*	N400: T > F
					T vs. S							*bobo-BOBA* vs. *caldo-SOPA*	P (250–350 ms): T > S; N400: T > S
Domínguez et al. (2006)	S	v	300	LD	Unrelated vs. related			Prefix-related *camello-REFORMA* vs. *reacción-REFORMA*	P (150–250 ms)	Syllable-related *camello-REFORMA* vs. *regalo-REFORMA*	No effect
					Pre vs. Syl						ant. N400: Pre > Syl
Smolka et al. (2015)	G	v	300	LD	Unrelated vs. related	Transparent TARNEN-ziehen vs. ZUZIEHEN-ziehen	N250, P325, N400			Form-related TARNEN-ziehen vs. ZIELEN-ziehen	(early) ant. P, N250, N400	Semantic TARNEN-ziehen vs. ZERREN-ziehen	N400
						Opaque *TARNEN-ziehen* vs. *ERZIEHEN-ziehen*	N250, P325, N400
					T vs. O		N400: T = O
					O vs. F					*ERZIEHEN-ziehen* vs. *ZIELEN-ziehen*	N400: O > F
					T vs. S							*ZUZIEHEN-ziehen* vs. *ZERREN-ziehen*	(early) P, N400: T > S
Kielar and Joanisse (2011)	E	a	500	LD	Unrelated vs. related	Transparent *illness-HUNT* vs. *hunter-HUNT*	N400	†Pseudo/opaque *message-CORN* vs. *corner-CORN*	No effect	Form-related *dragon-PLAN* vs. *planet-PLAN*	No effect	Semantic *doctor-BOX* vs. *carton-BOX*	No effect
						Transparent (semi) *dresser-CARE* vs. *careful-CARE*	N400
					T vs. Ts vs. P	*hunter-HUNT* vs. *dresser-DRESS* vs. *corner- CORN*	N400: T = Ts > P

Notes. All of the above studies used an immediate priming technique with visual (v) or auditory (a) prime presentation (PP) followed by visually presented targets to which participants made lexical decisions (LD) or semantic categorizations (SC). Visual primes were presented with #forward or backward# masks or unmasked with a stimulus onset asynchrony (SOA in ms) relative to the visual prime onset or the auditory prime offset. Languages (Lang) were E = English, G = German, and S = Spanish; Priming Conditions: I = identity, T = semantically transparent, Ts = semi-transparent, Pseudocomplex = pseudo-morphemic, O = semantically opaque, SHG = stem homograph, F = form-related, S = semantically related, U = unrelated, † 31/47 prime-target pairs were real morphological derivations but semantically opaque of the type *apartment-apart*, 16/47 were pseudocomplex of the *corner-corn* type; primes are presented first, targets second, in UPPER or lower case letters as in the corresponding study. ERP Effects: N = negativity, P = positivity, ant. = anterior, post. = posterior, = or ≠ indicates similar or different effects or > the size of the priming effect (i.e., with a more positive-going attenuation), *when corrected for removed items or multiple comparisons, ** = nonsignificant effects with significant linear trend between conditions; more positive-going deflections of related conditions relative to more negative-going unrelated conditions are interpreted as N250, if they occur within a negativity in the 200–300 ms post-target window, otherwise we refer to them as “early positivity”; Priming is concluded, if the negative going ERP amplitude in the latency range of 250 ms (N250) and 400 ms (N400) is attenuated relative to an unrelated baseline condition, in which the largest negative going amplitude is evoked.

ERP Studies using the masked visual priming paradigm with short prime presentations (below 50 ms) observed that real morphologically related (semantically transparent or identical) word pairs like *hunter-hunt* or *table-table* induced either an N250 attenuation alone (cf. Morris et al., 2008) or both N250 and N400 attenuations relative to the unrelated condition (cf. Holcomb and Grainger, 2006; Lavric et al., 2007; Morris et al., 2007, 2008, 2011, 2013). The variation of effects was more diverse regarding pseudocomplex word or nonword pairs of the *corner-corn* or *cornity-corn type or regarding form-related word pairs of the *scandal-scan* or *teble-table type, ranging from no effect in either condition (Morris et al., 2007), to N250 attenuations in both conditions (cf. Morris et al., 2008), to N250 alongside N400 attenuations in form-related pairs (cf. Holcomb and Grainger, 2006) or in both the pseudocomplex and form-related pairs (cf. Lavric et al., 2007; Morris et al., 2008, 2011, 2013).

Different results were obtained when the priming for morphologically complex words was compared with that of pseudocomplex or form-related words. While Morris et al. (2007) observed significantly more priming from morphologically related words than by either pseudocomplex or form-related words in both the N250 and N400 latency range, other studies by Morris et al. (2008, 2011, 2013) found no priming differences between these three types of complexity. Other studies, yet, revealed differential processing patterns during the early (N250) and later (N400) negativity. The similar N250 deflections by real morphologically and pseudomorphologically related word pairs were taken as evidence that all words undergo the same segmentation process in early visual word recognition. Similar N400 effects of pseudocomplex words and real complex words (Lavric et al., 2007; Morris et al., 2011) were interpreted to indicate a single mechanism with two-stages (orthography-based morphological decomposition followed by semantic interpretation, (see e.g., Meunier and Longtin, 2007; Lavric et al., 2011). By contrast, similar N400 effects of pseudocomplex and form-related words (Morris et al., 2008, 2011) were interpreted as evidence for a dual-route model that comprises two-mechanisms of decomposition (one orthography-based plus one semantically based, (see e.g., Diependaele et al., 2005; Holcomb and Grainger, 2006; Morris et al., 2013)).

Since the present study focuses on lexical representations (and not on early visual word recognition), we are indifferent with respect to models on early visual word recognition. Most importantly, all models so far assume different processing outcomes for semantically transparent and opaque words at the lexical level, when semantic information is integrated (in the two-stage model, e.g., Lavric et al., 2011), or when shared representations operate at the morpho-semantic level (in the dual-route model, e.g., Morris et al., 2013), or when form and meaning codes overlap (in the connectionist model, e.g., Plaut and Gonnerman, 2000). We will now turn to review the ERP studies that examined lexical representation and processing.

Under overt priming conditions with either auditory or visual prime presentations at long SOAs all the studies reviewed here observed N400 attenuations relative to the unrelated baseline for morphologically related and semantically transparent or inflected word pairs like *hunter-hunt* or *loca-loco* (“crazy woman”-“crazy man”), respectively (cf. Barber et al., 2002; Domínguez et al., 2004; Kielar and Joanisse, 2011; Lavric et al., 2011), as well as an additional early positivity for inflected word pairs (Domínguez et al., 2004). The picture was again, more diverse for pseudocomplex word-pairs of the *corner-corn* type or stem homographs of the *rata-rato* (“rat”-“time”) type, ranging from no effect at all for pseudocomplex words (Kielar and Joanisse, 2011), to an early positivity for stem homographs (Domínguez et al., 2004), to N400 attenuations for pseudocomplex words or stem homographs (cf. Barber et al., 2002; Domínguez et al., 2004; Lavric et al., 2011), and an additional modulation of a late negativity for stem homographs (cf. Barber et al., 2002; Domínguez et al., 2004). In contrast to pseudocomplex words, purely form-related words did not reveal substantial effects relative to the unrelated condition (cf. Domínguez et al., 2004; Kielar and Joanisse, 2011), though an N400 attenuation was found as well (cf. Lavric et al., 2011).

The main interest of the above studies was to investigate the processing of different levels of word complexity. For example, Lavric et al. (2011) found that the N400 effect was largest when it was induced by morphologically related word pairs like *hunter-hunt*, smaller by pseudocomplex words like *corner-corn* and smallest by purely form-related words like *brothel-broth*. They interpreted the differences in deflections in favor of a two-stage model of visual word recognition, with orthography-based morphological decomposition in the first stage, and validation by semantic information at a later stage.

By contrast, Kielar and Joanisse (2011) found evidence in favor of their convergence of codes view: They manipulated the semantic transparency between real morphological derivations by constructing a fully transparent condition of the *government-govern* type, a semi-transparent condition of the *dresser-dress* type, and a semantically opaque condition that comprised about two thirds real morphological derivations of the *apartment-apart* type and one third pseudomorphological derivations of the *corner-corn* type. They found similar N400 priming effects for semantically transparent and semi-transparent and no effect at all for semantically opaque pairs. In line with the distributed-connectionist or convergence of codes view “morphological effects were graded in nature and modulated by phonological and semantic factors” (Kielar and Joanisse, 2011, p. 170). Since neither pure form similarity like *panel-pan* nor semantic associations like *sofa-coach* produced any significant effects, the authors concluded that the morphological effects could not be explained by pure form or meaning relatedness alone.

### Lexical representation in German

To summarize, previous studies on lexical representation (using auditory or visual prime presentation at long SOAs) in English or French observed that semantic transparency plays a key role in lexical representation. These findings strongly contrast with our behavioral findings in German (Smolka et al., 2009, 2014): Under overt priming with either auditory or visual prime presentation (at long SOAs) complex verbs primed their base to the same extent regardless of whether they were semantically transparent (e.g., *aufstehen-stehen*, “stand up”-“stand”) or semantically opaque (e.g., *verstehen-stehen*, “understand”-“stand”). Unlike the English and French findings, these findings suggest that lexical representation in German is independent of semantic compositionality: A complex verb like *understand* is not only segmented into {under} and {stand} during early visual (or auditory) word recognition but is also lexically represented via its base {stand}.

Given that there are hardly any studies of this issue in German, we seek to investigate it more fully by means of ERPs. Behavioral responses reflect the endpoint of multiple stages of the word recognition process as well as response preparation. ERPs provide the possibility to tap online into the different processes of morphological, semantic, and form-relatedness—all processes that are hard to detect by means of purely behavioral priming techniques.

The present study used German complex verbs to examine whether morphologically complex words are lexically represented via their base or via their whole form. German complex verbs provide the opportunity to manipulate the semantic transparency and opacity relating to the same base verb. For example, the complex verbs *ankommen* (“arrive”), *mitkommen* (“come along”), *zurückkommen* (“come back”), *nachkommen* (“follow”), *entkommen* (“escape”), *abkommen* (“digress”), *bekommen* (“get”), *verkommen* (“degenerate”), and *umkommen* (“perish”) generate a wide range of meaning variation with respect to the same base verb *kommen* (“come”).

The linguistic literature (e.g., Fleischer and Barz, 1992; Olsen, 1996) distinguishes between prefix and particle verbs. Both types comprise a simple verb and a prefix or a particle. Prefix and particle verbs differ in some syntactic and prosodic characteristics. However, these differences do not surface under the stimulus presentation of the present study (i.e., in citation form under visual presentation). Further, previous studies (using phoneme monitoring or priming) found similar effects by prefix and particle verbs in German (Drews et al., unpublished) and Dutch (Schriefers et al., 1991). Hence, the two types are not further differentiated here and subsumed under the general term “complex verbs”.

Most importantly, prefix and particle verbs may be both transparently and opaquely related to the meaning of a base verb. For example, the prefix *ver-* occurs in the transparent prefix verb *verbleiben* (“remain”) of the base verb *bleiben* (“stay”), and in the opaque derivation *verschwimmen* (“blur”) of the base *schwimmen* (“swim”). Similarly, the particle *auf* (“up”) may occur in a transparent derivation like *aufheben* (“lift”) of the base verb *heben* (“lift”) as well as in an opaque derivation like *aufhören* (“stop”) of the verb *hören* (“hear”).

Importantly, and different from previous studies in English (e.g., Rastle et al., 2000, 2004; Morris et al., 2007), all complex verbs used in this study share a morphological relationship established on etymological grounds with their base, even if they are not semantically compositional.

### The present study

The present study is closely modeled on the behavioral study of Smolka et al. (2009). In that study, response latencies and accuracies were measured under overt priming conditions with visual prime presentations at long SOAs (300 ms) to ensure that the experimental conditions were sensitive to semantic processing and tapped into lexical processing. Morphologically related primes were complex verb derivations that were either transparently (e.g., *mitkommen*, “come along”) or opaquely (e.g, *umkommen*, “perish”) related to their base verb (e.g, *kommen*, “come”) that served as target. Contrary to the view that semantic meaning presides over conscious word processing, semantic transparency did not modulate the magnitude of morphological priming. In the first experiment, semantically transparent (*mitkommen*, “come along”), opaque (*umkommen*, “perish”), and identity (*kommen*, “come”) primes facilitated the recognition of base verbs (*kommen*, “come”) to the same extent. By contrast, purely semantically associated verbs (*nahen*, “approach”) did not prime.

The second experiment examined the influence of form overlap on morphological processing and exchanged the identical primes with form-related primes (*kämmen*, “comb”). However, form relatedness hindered target recognition (*kommen*, “come”) at the same time as morphological relatedness facilitated target recognition, again regardless of semantic transparency. In that experiment, semantic associates (*nahen*, “approach”) induced significant priming, though weaker in magnitude than that by morphologically related primes. In the third experiment, this time under prime-exposure durations of 1000 ms, priming from semantic associates was as strong as that by morphologically related primes; and accuracy (but not latency) data showed a small semantic transparency effect in favor of semantically transparent over opaque derivations.

To summarize, the three experiments demonstrated strong morphological priming that was (a) equivalent for semantically transparent and opaque complex verbs; (b) stronger than semantic priming (at SOA 300); and (c) different from form inhibition. These results were in line with previous behavioral experiments in German that observed equivalent priming from semantically transparent and opaque primes under overt priming conditions (e.g., Drews et al., unpublished; Schirmeier et al., 2004).

In the present study, we modeled on the second experiment of Smolka et al. (2009) and measured ERPs under overt visual priming conditions at 300 ms SOA. Priming was measured against the control condition with unrelated verb pairs like *tarnen-ziehen* (“mask”-“pull”). This condition was expected to yield the most negative potentials. As described above, we concluded priming, if the ERP amplitude of a related condition was attenuated (in the latency range around 250 ms or around 400 ms) relative to this baseline condition, which shows neither form nor semantic relatedness between primes and targets. A condition with semantic associations between verb pairs like *zerren-ziehen* (“drag”-“pull”) was used to measure the pure meaning relatedness between verbs. We hypothesized that this condition will induce an N400 attenuation, if semantic associations between verbs are strong enough to activate the automatic spreading within a semantic network. Looking at previous results (see Table 1) it is possible though, that semantic associations do not induce significant effects (cf. Kielar and Joanisse, 2011), while synonyms do (cf. Domínguez et al., 2004).

We induced morphological priming by using real morphological derivations that were either semantically transparent or opaque with respect to their base, such as *zuziehen-ziehen* (“pull together”-“pull”) and *erziehen-ziehen* (“educate”-“pull”), respectively. As in all previous ERP studies using overt priming (Barber et al., 2002; Domínguez et al., 2004; Kielar and Joanisse, 2011; Lavric et al., 2011), we expected a strong N400 attenuation for semantically transparent derivations.

With respect to the semantically opaque condition, this is the first ERP study that used semantically opaque words that were real morphological derivations of their base. There is only one comparable study that used (partly) real morphological but semantically opaque derivations, and they did not find any priming effect in this condition (Kielar and Joanisse, 2011). According to the two-stage model or dual route view that morphological structure and processing depends on semantic compositionality (i.e., the meaning relation between prime and target), we should not find any effect for semantically opaque words. If, however, German complex words are accessed and represented via their stem regardless of meaning compositionality, we will observe a priming effect in form of an N400 attenuation in this condition, too.

Similarly the connectionist or convergence of codes view assumes that semantic similarity plays a role in that “morphological effects vary continuously as a function of the degree of semantic and form similarity among words” (Kielar and Joanisse, 2011, p. 162). In the present study, the morphologically related primes all contain the complete target and thus share the same form overlap between prime and target. Hence, the only difference between morphologically related primes in the semantically transparent and opaque conditions is that the former have a strong meaning similarity with the target, while the latter show no or only little meaning similarity with the target. Semantically opaque words should thus induce either no priming at all—as it was the case in the behavioral study by Gonnerman et al. (2007) and in the ERP study by Kielar and Joanisse (2011)—or, in case that semantically opaque words do induce priming, its magnitude should be significantly weaker than that by transparent words. In ERP terms, if the morphological effects were a combination of form and meaning overlap, we should find stronger effects, that is, more positive-going N400 amplitudes, for semantically transparent than for semantically opaque primes. If, however, our view holds that all German complex words access and activate their base, and if our previous behavioral findings (Smolka et al., 2009, 2014) generalize to electrophysiological data, we will find equivalent priming effects by semantically transparent and opaque derivations. Additionally, if our hypothesis holds that morphological regularities generalize beyond meaning relatedness, we should expect stronger morphological than semantic effects. In ERP terms, the N400 effects will be more positive-going for the two morphological conditions than for the semantic condition.

Morphologically related pairs are always form-related as well. Hence, orthographically similar verbs like *zielen-ziehen* (“aim”-“pull”) were used to measure the effects of form similarity between verbs. Previous overt priming studies so far revealed either a very small form effect (cf. Lavric et al., 2011) or none at all (Domínguez et al., 2004, 2006; Kielar and Joanisse, 2011). For this reason, we were indifferent as to whether we should expect a significant form effect relative to the baseline condition. Importantly, though, if our hypothesis holds that morphological structure in German generalizes beyond form, the amplitude in the form condition will be more similar to the unrelated condition and hence more negative than that of the morphologically related but semantically opaque condition (both representing form without meaning relatedness).

## Methods

### Participants

Seventeen students of the Philipps-University, Marburg, took part in the experiment for course credit or payment. All participants were monolingual speakers of German, not dyslexic and had normal or corrected-to-normal vision. All participants were right-handed and gave their written informed consent.

### Materials

#### Critical stimuli

Thirty-six different base verbs were selected as critical targets; each target was combined with five primes. Table 2 summarizes the stimulus characteristics of primes and targets (for the whole stimulus set, see Smolka et al., 2009). For illustration, we consider the example *ziehen* (“pull”). Three factors defined the prime-target relations: morphological, semantic, and form relatedness with the base verb. All morphological derivations held a prefix or particle and were, by definition form-related to the target: (a) T, semantically transparent derivations of the base like *zuziehen* (“pull together”); (b) O, semantically opaque derivations of the base like *erziehen* (“educate”). All other primes comprised simple verbs that were morphologically unrelated to the target; (c) S, purely semantically related verbs like *zerren* (“drag”); (d) F, form-related verbs like *zielen* (“aim”) where the onset or first syllable matched that of the target and where the rime differed from the target by a single grapheme (1 or 2 letters); all but two form-related primes were verbs; (e) U, unrelated verbs like *tarnen* (“mask”) were neither semantically nor form related to the target.

**Table 2:** **Stimulus characteristics of primes that were semantically related (S), morphologically related and semantically transparent (T), morphologically related and semantically opaque (O), form-related (F), or unrelated (U) to targets**.

	Lemma frequency	Word form frequency	Number of letters	Relatedness score
**Target**	355.2 (431)	98.1 (128)	6.6 (1.3)	
*ziehen* (“pull”)
**S**	143.8 (328)	40.2 (81)	6.7 (1.4)	5.9 (0.6)
*zerren* (“drag”)
**T**	11.7 (19)	2.3 (4)	10.1 (2.1)	5.1 (0.7)
*zuziehen* (“pull together”)
**O**	17.6 (32)	3.4 (6)	9.6 (1.5)	2.8 (0.6)
*erziehen* (“educate”)
**F**	29.3 (70)	7.6 (19)	6.9 (1.2)	1.8 (0.6)
*zielen* (“aim”)
**U**	15.4 (18)	3.0 (6)	6.6 (1.0)	1.4 (0.3)
*tarnen* (“mask”)

*Note. Mean lemma and word form frequencies, mean number of letters and mean rating scores (on a 7-point scale from 1 to 7); standard deviations in parentheses. All frequencies are from the CELEX database (Baayen et al., 1993), count is per million*.

Semantically associated prime-target pairs had a (position-specific) mean letter overlap of 20% (SD = 17); form-related primes had a letter overlap of 70% (SD = 23) with the targets, and unrelated prime-target pairs shared 10% (SD = 13) of the letters. The primes of the two morphologically related conditions, by definition, contained the whole target words.

The meaning relatedness between primes and targets was tested in a previously conducted association test (for details, see Smolka et al., 2009). The five prime conditions of the same target were distributed across five lists according to a Latin square design. Fifty native speakers of German (who did not participate in the ERP experiment) rated on a 7-point scale from *completely unrelated* (1) to *highly related* (7) whether two verbs like *erziehen-ziehen* are meaning related.

The verb pairs in the S and T conditions were rated as being highly semantically related with mean ratings of 5.9 (SD = 0.63) and 5.1 (SD = 0.68), respectively. By contrast, verb pairs in the O, F, and U conditions were rated low in meaning relatedness with mean ratings of 2.8 (SD = 0.66), 1.8 (SD = 0.65), and 1.4 (SD = 0.35), respectively. A one-way ANOVA was performed on mean rating scores with items (*F*_2_) as random variables. The repeated measures factor Prime Type (S/T/O/F/U) was highly significant, *F*_2_(4, 139) = 414.03, *p* < 0.0001. Scheffé *post hoc* comparisons confirmed that the mean rating scores of the S, T, and O conditions significantly differed from each other as well as from the F and U conditions, while the latter two did not significantly differ. Most importantly, with respect to the morphological conditions, semantically transparent primes like *zuziehen* (“pull together”) were rated as significantly higher (5.1) related in meaning to their target base *ziehen* (“pull”) than semantically opaque (2.8) primes like *erziehen* (“educate”).

#### Filler prime-target pairs

Five hundred and forty filler pairs were added to the 180 critical pairs. All filler pairs were semantically unrelated and differed from the items of the critical set. All filler pairs had words as primes, which followed the same morphological composition as the experimental set: 216 primes were complex verbs and 324 were simple verbs.

Similar to the critical set, 180 filler pairs had pseudoverbs as targets that were form-related to the 36 base verbs of the critical set. All pseudoverbs followed the phonotactic constraints of the German language. For example, the pseudoverbs *stehmen, *stehnen, *steben, *steken, and *stedern were created to be form-related to the verb *stehlen*. The rest of the filler pairs had 180 verb targets and 180 pseudoverb targets.

#### Summary of the stimulus material

To summarize, the whole material set comprised 720 prime-target pairs. Half of them had verbs, the other half had pseudoverbs as targets. Primes were always existing verbs: 288 (40%) were complex verbs and 432 (60%) were simple verbs. All primes and targets were presented in the infinitive (stem + -*en*), which is also the citation form in German.

The large amount of fillers should diminish both facilitatory and inhibitory effects (Napps and Fowler, 1987) and prevent both expectancy and failed expectancy effects. Overall, the proportion of (a) critical prime-target pairs was reduced to 25%; (b) that of semantically related pairs to 10%; and (c) that of form-related prime-target pairs (both words and nonwords) to 15% of the entire material.

### Apparatus

Stimuli were presented on a 17″ monitor, connected to an IBM-compatible personal computer. Stimulus presentation and data collection were controlled by the *Presentation* software developed by Neurobehavioral Systems.^2^ Responses were recorded from the left and right “control” keys on a standard keyboard.

### Design

Each participant saw all 36 simple verbs in all five priming conditions. Primes of the same target were rotated over ten blocks according to a Latin Square design, in such a way that the same target appeared in every second block. Likewise, the prime-target pairs of similar pseudoverb targets were distributed across the ten blocks. The remaining filler pairs were evenly allocated to the blocks, so that each block comprised equal numbers of complex and simple primes as well as verb and pseudoverb targets.

In total, an experimental session comprised 720 prime-target pairs, presented in ten experimental blocks, with 72 prime-target pairs per block. Within blocks, prime-target pairs were randomized separately for each participant with the constraint that there were maximal four adjacent word or nonword targets. There were 20 practice trials.

### Procedure

Participants were tested individually in a dimly lit room and were seated at a viewing distance of about 60 cm from the screen. Each trial started with a fixation cross in the center of the screen for 1000 ms. Primes and targets were presented in the center of the screen, in white Sans Serif letters on a black background. Primes were presented in uppercase letters, point 22, targets in lowercase letters, point 26. The prime appeared for 200 ms, followed by a blank screen for 100 ms (SOA = 300 ms), after which the target appeared for 500 ms. A prompt (*“?”*) appeared 1000 ms after target-onset on the screen. The inter-trial interval was constant at 2000 ms.

Participants were asked to refrain from blinking and to respond until after the prompt. Participants made lexical decisions to the targets, in other words, they responded whether the stimuli were existing words or not, and were instructed to respond as accurately as possible.^3^ “Word” responses were made by pressing the right “control” keyboard key with the index finger of the right hand, “nonword” responses were made with the left hand on the left “control” key. During practice trials, participants received feedback on the accuracy of each response; during the experimental session, feedback was given only on incorrect responses.

The experiment lasted for about 1 h. Participants self-administered the breaks between the ten blocks, and took at least two longer breaks.

### EEG recording

**Figure 1:**
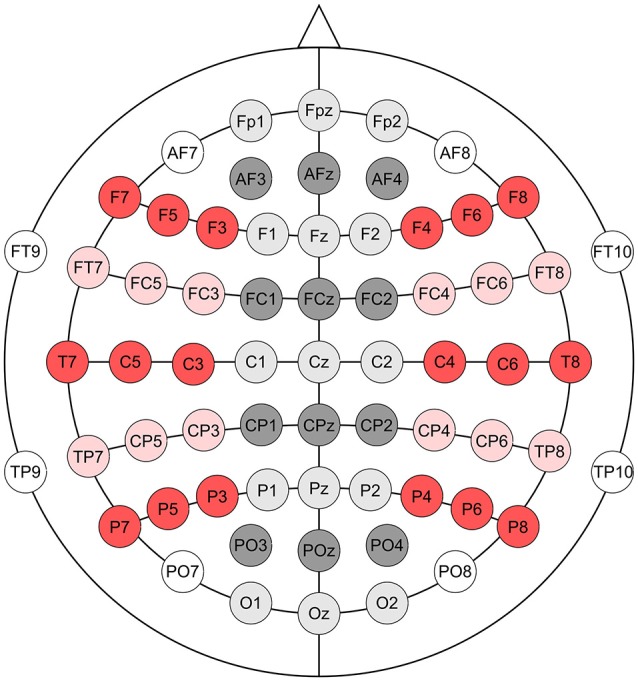
**Electrode montage**. Nineteen pooled electrodes, corresponding to the 19 electrodes of the 10–20 system were used in the analyses of the EEG data. Each of the pooled electrodes comprised three adjacent electrodes, as follows: Fpz (Fp1, Fpz, Fp2), AFz (AF3, AFz, AF4), F5 (F3, F5, F7), Fz (F1, Fz, F2), F6 (F4, F6, F8), FC5 (FC3, FC5, FT7), FCz (FC1, FCz, FC2), FC6 (FC4, FC6, FT8), C5 (C3, C5, T7), Cz (C1, Cz, C2), C6 (C4, C6, T8), CP5 (CP3, CP5, TP7), CPz (CP1, CPz, CP2), CP6 (CP4, CP6, TP8), P5 (P3, P5, P7), Pz (P1, Pz, P2), P6 (P4, P6, P8), POz (PO3, POz, PO4), Oz (O1, Oz, O2).

The EEG was recorded from 61 scalp electrodes using a cap in which Ag/AgCl inserts are fixated by individual electrode supports (System Falk Minow, Munich, Germany). All scalp electrodes were randomly referenced to the left or right earlobe during the recording and re-referenced offline to averaged earlobes; the left or right mastoid served as ground. Horizontal and vertical eye movements were monitored with appropriate electrode pairs. Impedances of all electrodes were kept below 5 kΩ. Two 32-channel amplifiers (SYNAMPS, NeuroScan) were used for EEG recording. Band pass was set from DC to 40 Hz and the sampling rate was 500 Hz. Prior to the beginning of each experimental block, a DC reset was manually initiated. DC drift was corrected according to the method suggested by Hennighausen et al. (1993). Eye blinks and trials with other artifacts were removed by applying a threshold criterion (max. voltage step per sampling point >50 μV or absolute difference in a trial segment >100 μV). ERPs were extracted from the edited set of raw data by averaging single trials separately for subjects, electrodes, and experimental conditions. Post-stimulus epochs were baseline-adjusted to the average amplitude of a 100 ms epoch preceding the onset of the target word. Only segments with correct responses entered the analysis. We created a subset of electrodes resembling the 19 standard electrodes of the 10–20-system. Three adjacent electrodes were pooled for each of these “standard” electrodes (see Figure 1). The pooled 19 electrodes entered the statistical analysis.

### Analysis of event-related potentials

Semantic priming effects were investigated to assess the sensitivity of the experimental setup. For this purpose, the ERPs to targets with semantically related primes were compared to unrelated primes (S vs. U) using standard *ad hoc* methods for ERP comparisons of two conditions. Semantic priming effects were assumed at those electrodes and time intervals at which S differed from U in pointwise *t* tests (*α* = 5% two-tailed) for an interval of at least 50 ms. Similar *ad hoc* analyses were performed to assess the influence of morphology (O vs. U) and form (F vs. U). The electrodes and locations at which semantic priming effects were observed were then used to define a region of interest (ROI) for the analyses of the interplay between semantic and morphological priming.

Permutation tests were used to assess whether these *ad hoc* methods yielded robust results (Blair and Karniski, 1993). Permutation tests allow controlling the family-wise Type 1 error rate in multiple, possibly dependent significance tests (for a review, see Maris and Oostenveld, 2007). To this end we calculated a *t*_max_ distribution for S – U using 10,000 permutations. In each permutation, the sign of S – U was selected at random for each participant, thereby simulating the null hypothesis in which *x* = S – U has the same probability than −*x* = U – S. We calculated the *t* values at each sampling point that fell into the ROI, and chose the maximum absolute value over all electrode clusters and time points (*t*_max_). The 95th percentile of this permutation distribution was selected as the critical *t*_max_. This means that the probability is 5% that any absolute *t*_max_ value in the main analysis is above the critical *t*_max_ value if the null hypothesis holds. Similar procedures were applied for investigating morphological and form-related priming.

The primary question of this study is whether semantic transparency exerts priming on top of morphological priming (T vs. O). In order to avoid overly conservative correction due to the high number of partial tests in the permutation procedure, the permutation test that examined T – O was restricted to a ROI that was defined on the basis of the standard semantic priming effect, obtained by the comparison of semantically related to semantically unrelated primes, S – U. The ROI included those electrodes and time intervals at which S – U differed from zero in running *t* tests (α = 5% two-tailed) for at least 50 ms. Such a ROI approach is able to control the Type 1 error while preserving power: For electrodes and intervals where semantic priming effects are observed, that is, S ≠ U, it can be expected that T differs from O to a similar extent, if the same processes of semantic analyses are activated. Since we are interested in the differential effect of semantic priming on top of morphology, we will be testing the difference between T and O only at electrodes and intervals where semantic priming effects are visible (e.g., Gondan et al., 2007). Excluding other time points and electrodes from the analysis thereby reduces the set of partial tests that have to be controlled. The critical *t*_max_ value is equally reduced, thereby increasing power to detect priming effects within the ROI.

## Results

### Semantic priming

**Figure 2:**
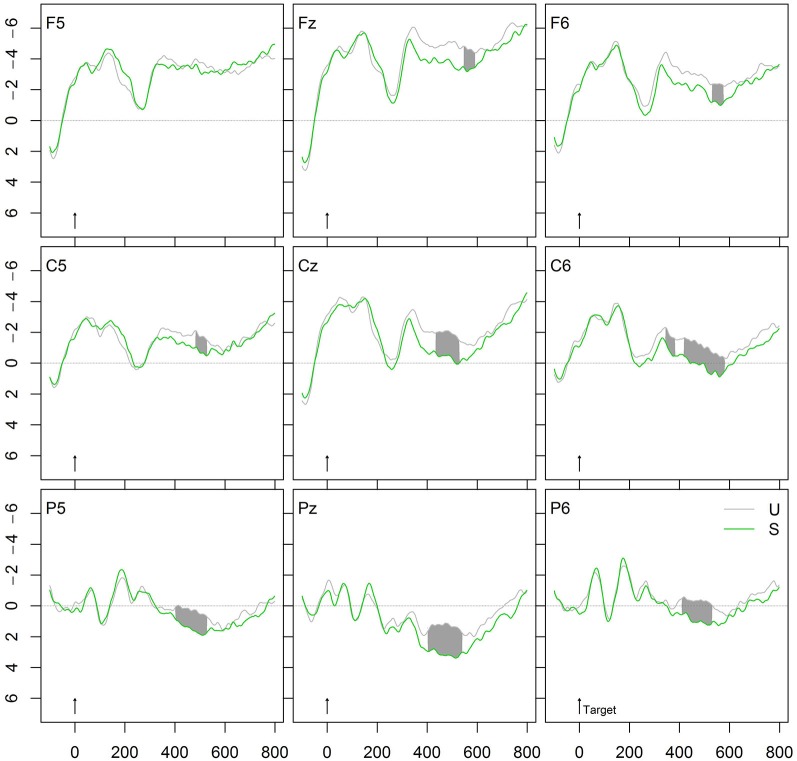
**Grand average ERPs of verb targets preceded by unrelated (U) or semantically related (S) verbs**. In this and the following figures, negativity is plotted upwards, with time in ms and potentials in μV.

Figure 2 shows the grand averages of semantically related (S, *zerren-ziehen*, “drag”-“pull”) and unrelated (U, *tarnen-ziehen*, “mask”-“pull”) prime-target pairs. The curves start to deviate from each other at about 300 ms after stimulus onset with unrelated targets being more negative than associated targets on the central and posterior electrodes. The maximum difference is reached around 400–600 ms, indicating the typical attenuation of the N400 component by semantic associations. The upper panel in Figure 6 provides the significant *t*- and permutation tests for this semantic effect.

**Figure 3:**
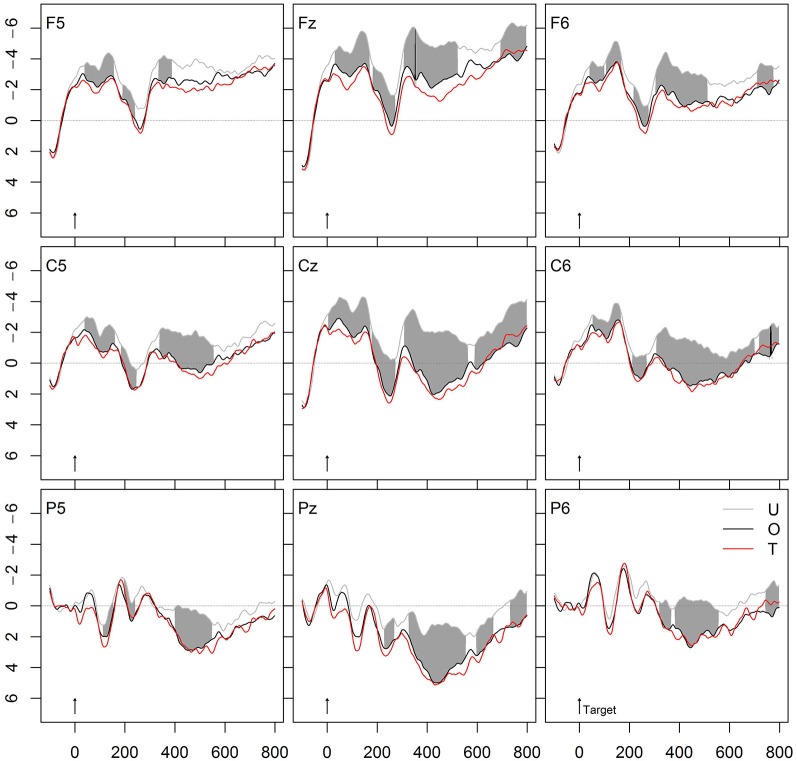
**Grand average ERPs of verb targets preceded by unrelated verbs (U) or morphologically related and semantically transparent (T) or morphologically related and semantically opaque (O) derivations**.

**Figure 4:**
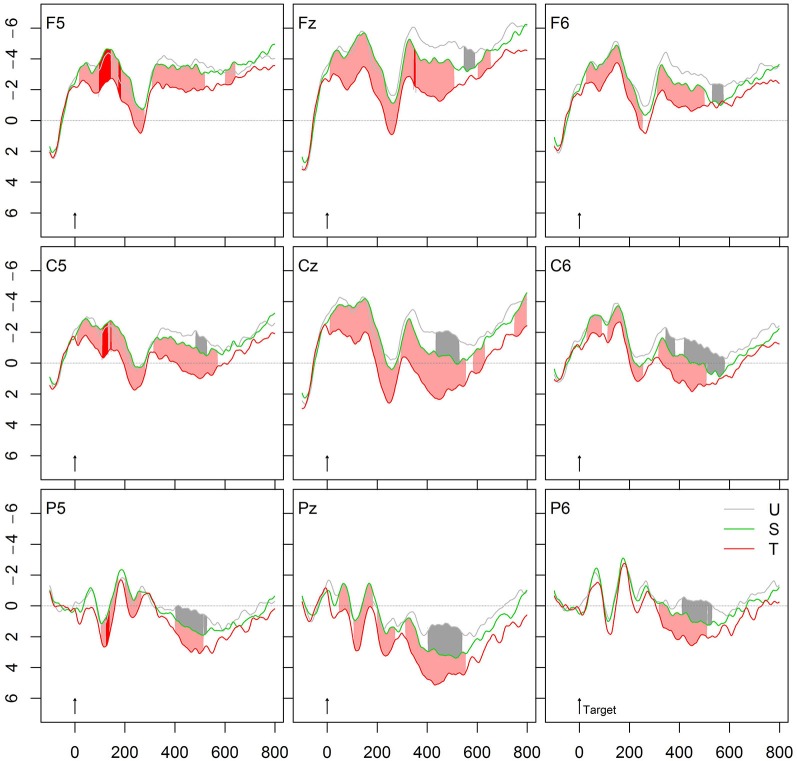
**Grand average ERPs of verb targets primed by unrelated (U), semantically related (S) verbs or morphologically and semantically transparent verbs (T)**.

**Figure 5:**
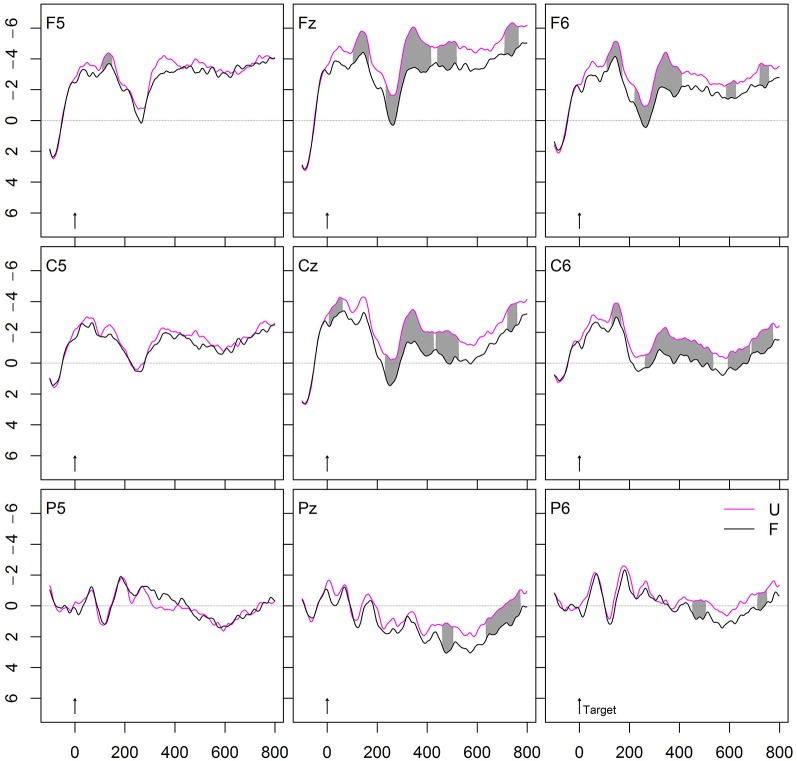
**Grand average ERPs of verb targets preceded by unrelated (U) or form-related (F) verbs**.

**Figure 6:**
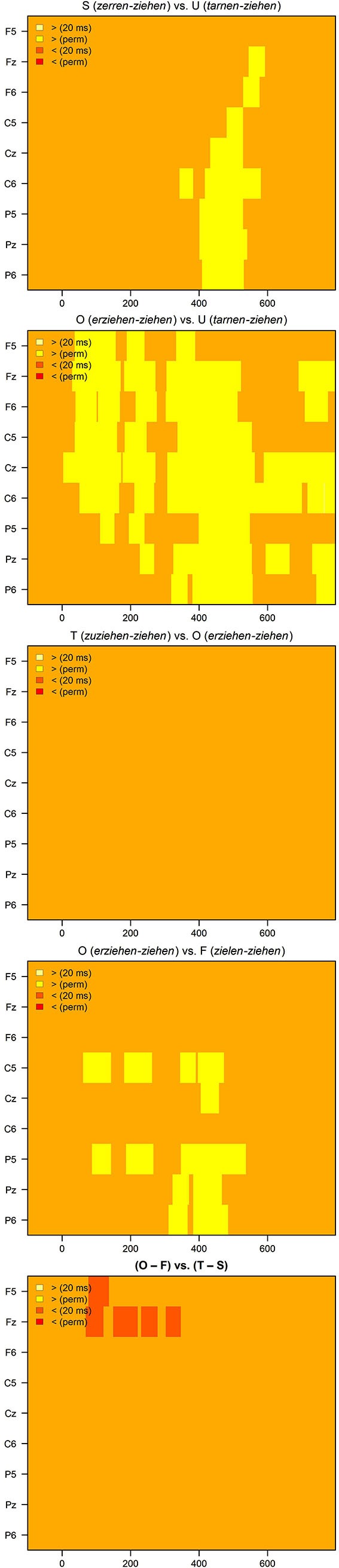
**The significance of *t*- and permutation tests from top to bottom: S vs. U, O vs. U; T vs. O; O vs. F; the difference O – F vs. the difference T – S**.

### Morphological priming

Do morphologically related complex verbs prime their base? To calculate the priming induced by morphological relatedness, both morphological conditions (T and O) were compared with the unrelated condition U. Figure 3 depicts the results. Each of the morphological conditions was far more positive going than the unrelated condition. The curves start to deviate in an early negativity, indicating an N250, followed by a positivity (P325), which again was followed by a strong N400 effect. The amplitude deviations between U and T as well as between U and O in the range of the N250, P325, and N400 components were significant for all electrode clusters and for both semantically transparent and opaque derivations. The second panel from the top in Figure 6 provides the significant *t*- and permutation tests for the pure morphological effect (O vs. U).

Is there a semantic transparency effect in the lexical representation of morphologically related German words? To this end, we compared the ERPs of morphologically and semantically transparent word pairs (T, *zuziehen-ziehen*, “pull together”-“pull”) to those of morphologically related, but semantically opaque word pairs (O, *erziehen-ziehen*, “educate”-“pull”). Figure 3 shows the striking similarity of the two conditions: Most importantly, the priming effects of T and O were equivalent in amplitude. In line with this, the permutation test within the ROI defined by S – U did not reveal any significant difference between T and O. The third panel from the top in Figure 6 demonstrates this resemblance between the two morphological conditions (T and O) in the *t*- and permutation tests.

We further tested the hypothesis whether or not morphological regularities generalize beyond meaning relatedness. If this is the case, we should find stronger morphological than semantic effects. Indeed, Figure 4 shows the comparison between the conditions S and T, and indicates that the effects induced by morphologically and semantically related prime-target pairs were much stronger than the N400 effect produced by pure semantic associations.

### Form priming

To calculate whether form-relatedness affects target recognition, a condition with orthographically similar verbs (F, *zielen-ziehen*, “aim”-“drag”) was compared with the unrelated baseline condition (U). As can be seen in Figure 5, this form effect starts at about 180 ms in a right frontal positivity (relative to the unrelated condition) that converges to an N250 effect and further extends to a weak frontal N400 effect. Form-related prime-target pairs typically induce the early positivity and N250 effects.

Importantly, this form effect significantly differed from the priming effect by morphologically related word pairs. While the form effect is right frontal, the morphological effect occurs at centro-parietal sites that characterize a typical N250 and N400 effect. The forth panel from the top in Figure 6 provides the significant *t*- and permutation tests comparing the two effects “form without meaning”, that is, O vs. F.

Finally, we calculated the comparison (O – F) vs. (T – S). This comparison represents the effects of form-relatedness (without meaning) with those of meaning relatedness. The effect occurs left anterior, with the difference (O – F) more negative going than the amplitude of the comparison (T – S), indicating an extended N250 effect or an anterior positivity. The bottom panel in Figure 6 provides the corresponding significant *t*- and permutation tests for this comparison.

## Discussion

This study investigated the lexical representation of German complex verbs and compared the processing of morphological derivations that were either semantically transparent or opaque with respect to their base. Since effects of semantic transparency and semantic association are difficult to detect in either the masked or the long-term priming task, we used immediate repetition priming; and since semantic effects among morphological relatives tend to increase with SOA (for a review, see Raveh and Rueckl, 2000; Feldman and Prostko, 2002; Feldman et al., 2004), we used overt visual prime presentations at 300 ms SOA. We thus made sure that we are tapping into lexical processing. Our results were straightforward: We observed strong morphological priming effects in both conditions. Before we discuss these effects in more detail, though, we will first turn to inspect the semantic and form effects.

### Semantic priming

As hypothesized, we found a broad N400 effect with attenuated curves for semantically related verbs (*zerren-ziehen*, “drag”-“pull”) relative to the unrelated verbs (*tarnen-ziehen*, “mask”-“pull”). This modulation of the N400 component is typical for semantic associations and it indicates that the semantic associations between verbs are strong enough to activate automatic spreading within a semantic network. In contrast to Kielar and Joanisse (2011) who observed no effect for semantic associations, our findings indicate that not only synonyms (cf. Domínguez et al., 2004) but also semantic associations are automatically activated within the semantic network.

Even though the N400 attenuation we found for semantic associations might be smaller than expected, one has to keep in mind that we are dealing with verb-verb pairs, which generally show smaller priming effects than noun-noun associations. While there are plenty of ERP studies measuring the effects of semantic association between nouns (e.g., Bentin et al., 1985), there are only few measuring the semantic relatedness between verbs (cf. Rösler et al., 2001; Smolka et al., 2013), so that there are only few studies for a direct comparison. With respect to verb pairs, we have repeatedly found a dissociation between the electrophysiological and the behavioral data: While the former always indicated strong semantic-priming effects in terms of N400 modulations (Rösler et al., 2001; Smolka et al., 2013), the latter generated both significant (cf. Exp. 2 and 3 in Smolka et al., 2009; Exp. 3 in Smolka et al., 2014) and nonsignificant priming effects (cf. Exp. 1 and 2 in Smolka et al., 2014; Exp. 1 in Smolka et al., 2009, 2013). This dissociation between electrophysiological and behavioral data suggests that ERPs represent a fine-grained means that makes it possible to measure subtle effects that do not surface under behavioral data collection.

Most importantly, the present N400 deflection by semantic associations proves that the experimental procedure in this experiment is sensitive to detecting semantic influences and tapping into lexical processing.

### Form priming

To control the effects of form similarity, we compared orthographically similar verbs like *zielen-ziehen* (“aim”-“drag”) with the unrelated baseline condition. Orthographically similar primes induced a priming effect in terms of an early right frontal positivity that converges into an N250 effect and further extends to a frontal N400 effect. Form-related prime-target pairs typically induce the early positivity and N250 effects. This finding corresponds to previous masked priming studies that found anterior N250 and N400 effects (Holcomb and Grainger, 2006; Lavric et al., 2007; Morris et al., 2008, 2011, 2013) as well as to an overt priming study that observed an N400 attenuation effect for form priming (Lavric et al., 2011).

The early positivity is typical for form-related relative to unrelated prime-target pairs. The dual-route model, for example, assumes two parallel mechanisms (one orthography-based and one semantically based). Form-priming in terms of the N250 reflects the mapping of prelexical representations onto whole-word representations (specifically, a feed-forward prelexical morpho-orthographic segmentation that operates independently of lexical status and semantic transparency, see Morris et al., 2011), while later (N400) effects are thought to indicate the mapping of shared representations at the morpho-semantic level (see e.g., Diependaele et al., 2005; Holcomb and Grainger, 2006; Morris et al., 2011, 2013). By contrast, the two stage-model assumes a single mechanism with two-stages, an orthography-based morphological decomposition followed by semantic interpretation (e.g., Meunier and Longtin, 2007; Lavric et al., 2011).

Most importantly, the form condition in our study was more negative going than the morphologically related but semantically opaque condition. Since both conditions represent form similarity without meaning relatedness, it is interesting to note that the comparison of the two generates an N400 attenuation, which is typical for semantic effects (with the morphological modulation being more positive than the form condition). This indicates that even semantically opaque but morphologically related pairs are more strongly meaning related to their base than purely form-related pairs are. We will discuss this issue in more detail in the description of the model below.

Overall, we may conclude that the morphological effects we obtained with German complex verbs cannot be reduced to pure semantic and form relatedness between words.

### Morphological priming

To examine whether morphologically related complex verbs prime their base, we compared the two morphological conditions relative to the unrelated condition. Both curves were far more positive going than the unrelated condition, each producing an N250, followed by a P325, again followed by a strong N400 attenuation effect at all electrode clusters.

The strong N400 attenuation for semantically transparent derivations corresponds to the findings of all previous ERP studies using overt priming (Barber et al., 2002; Domínguez et al., 2004; Kielar and Joanisse, 2011; Lavric et al., 2011). In addition, we also found a strong N400 attenuation for semantically opaque derivations, which contrasts with a previous study using (partly) real morphological but semantically opaque derivations, which did not find any priming in this condition (Kielar and Joanisse, 2011). Our findings thus indicate that German complex words are accessed and represented via their stem regardless of meaning compositionality.

Moreover, we observed not only a strong N400 modulation by semantically opaque derivations but also that this N400 attenuation was as strong as that by semantically transparent derivations. That is, *erziehen* (“educate”, semantically opaque) primed its base *ziehen* (“pull”) to the same extent as *zuziehen* (“pull together”, semantically transparent) did. This indicates that both derivations are accessed via their base regardless of their meaning relation to it.

The finding of equivalent priming from semantically transparent and opaque derivations corresponds to our previous behavioral findings (e.g., Smolka et al., 2014). Specifically, in the behavioral experiment using the same stimulus material and priming conditions as in this ERP study (Smolka et al., 2009), semantically transparent and opaque derivations yielded 43 ms and 40 ms priming effects, respectively (see also the summary of behavioral effects in Table 6 in Smolka et al., 2014). Altogether, these data indicate that semantically transparent and opaque derivations are lexically represented and processed in similar ways. We will discuss this issue in more detail in our proposed model of lexical representations (see below).

Finally, we asked whether morphological regularities generalize beyond meaning relatedness. Indeed, we found stronger N400 attenuation effects for morphological than semantic relatedness. This finding is particularly interesting, because the ratings of the association test indicated that semantic associates like *zerren* (“drag”) were rated as significantly higher (5.9) related in meaning to the target *ziehen* (“pull”) than the morphologically related and semantically transparent (5.1) primes like *zuziehen* (“pull together”).

Stronger priming for morphologically related and semantically transparent primes (i.e., in the T condition) than in the semantic condition can be readily explained by the convergence of codes view. Given that primes and targets in the T condition overlap both in form and meaning, the N400 should be more positive-going than with either orthographic (in the F condition) or semantic overlap (in the S condition) only. However, according to the same argument, the N400 amplitude in the O condition should be significantly less positive-going than in the T condition, since opaque primes share the form but no or little meaning with the target, but this was not the case.

With respect to the pure semantic effect, its occurrence is important since it indicates that the design of this study was sensitive to detecting semantic influences. The lack of semantic transparency effect in the morphological condition is thus not due to a general lack of semantic processing in this study.

**Figure 7:**
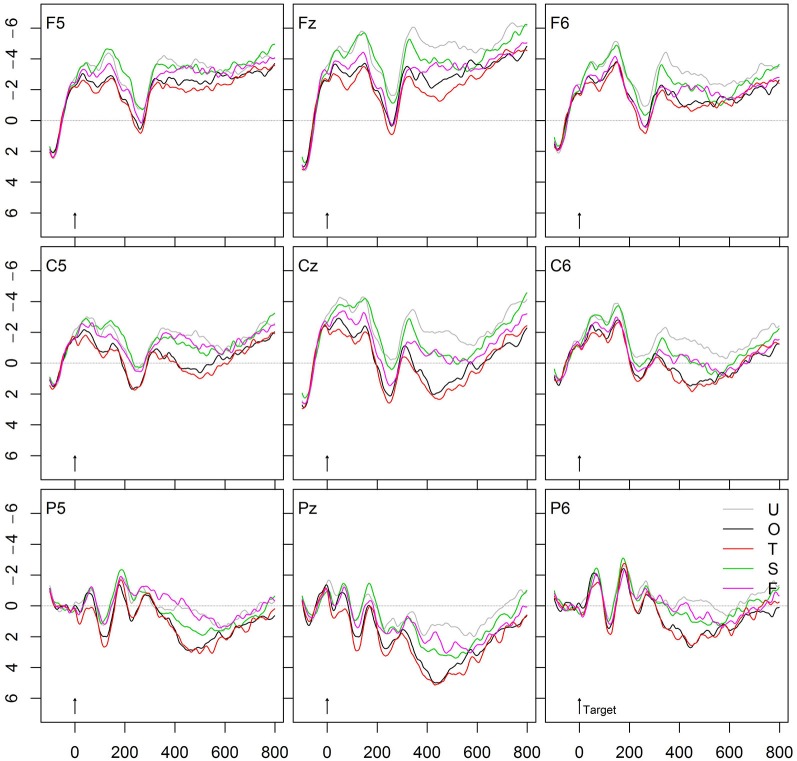
**Grand average ERPs of verb targets preceded by unrelated (U), form-related (F), semantically related (S), morphologically related and semantically transparent (T) or by morphologically related and semantically opaque (O) verbs**.

A direct comparison of the present ERP data and the corresponding RT data from the study, modeled on Smolka et al. (2009), reveals striking similarities. Figure 7 provides all conditions for an easy overview. Targets in the unrelated condition showed slow RTs (532 ms) and the most-negative going N400 amplitude. This condition served as the baseline against which the priming effects were calculated. Form-related primes significantly inhibited responses (+16 ms) in the behavioral data and induced slightly more positive-going N250 and N400 amplitudes as compared with the unrelated condition. By contrast, the semantic associates yielded faster RTs (−21 ms) and a more positive-going N400 amplitude than the unrelated condition. This semantic effect was smaller than the morphological effects, that is, RTs were slower (≈20 ms) and ERPs were more negative-going than in the morphological conditions. Further, the two morphological conditions, T and O, yielded the strongest priming effects relative to the unrelated condition. This was evident in terms of the fastest RTs (−43 ms and −40 ms, respectively) and the most positive-going N400 amplitudes. Most importantly, neither the RTs nor the ERPs differed between the two morphological conditions. Finally, the morphologically related but semantically opaque condition showed significantly faster RTs (−56 ms) and far more positive-going N400 amplitudes than the form condition.

If we summarize the behavioral (Smolka et al., 2009, 2014) and the electrophysiological data presented here, we may conclude that both types of data revealed strong morphological priming effects that were significantly larger than those induced by purely semantically related or form related complex verbs. This general convergence means that the morphological-priming mechanism involves a general cognitive phenomenon that can be captured by different methods. This renders our results even more robust: We have shown that complex verbs in German are accessed and processed via their stem, regardless of their meaning compositionality.

We thus provide evidence for the existence of a morphological dimension to lexical organization that cannot be reduced to formal or semantic relations between primes and targets. Most importantly, this indicates that morphological structure needs to be incorporated in the modeling of lexical representation in German.

Why is it that morphological processing and representation seems to be different in German compared to other Indo-European languages like English or French? In the following, we consider some possible factors that may affect language processing.

#### Affixation type

One might argue that the origin of the strong morphological effects (without effects of semantic transparency) in our study arose due to the use of prefixed (in contrast to suffixed) words. Indeed, only few overt priming studies (Marslen-Wilson et al., 1994; Feldman et al., 2002; Zwitserlood et al., 2005) used prefixed prime-target pairs that are similar to those in the present study. Nevertheless, they found priming from prefixed words only if they were semantically transparent (e.g., *disobey-obey* in English, *privole-volim* in Serbian, or *meebrengen-brengen* in Dutch), but not if they were semantically opaque (e.g., *restrain-strain*, *zavole-volim*, or *ombrengen-brengen*, respectively). Only prefixed verbs in German induced morphological priming from semantically opaque verbs (Drews et al., unpublished). We may thus conclude that the affixation type was not the critical factor that caused the morphological priming effects.

#### Productivity

The productivity of verb derivations in German is extremely high. A single base verb may yield families of up to 150 complex (prefix or particle) verbs, all with different meanings ranging from truly transparent to truly opaque. For example, the German base *stehen* (“stand”) has more than 100 prefixed derivations, while the same base *stand* in English possesses the prefixed derivations *understand* and *withstand* and about 20 phrasal verbs (cf. McCarthy et al., 2006). Furthermore, any complex verb is conjugated in exactly the same way as its base verb (i.e., with the same irregularities, if there are any) and thus keeps the link to its origin. Due to the high number of family members, German speakers may be more responsive to the base than English speakers are.

It is possible that the productivity of German verbs leads to a generalization of (morphological) form that becomes relatively independent of meaning relatedness, as it is the case in root languages like Hebrew and Arabic. Indeed, some connectionist accounts suggest that whether one finds morphological priming without meaning relatedness depends on the morphological structure of the language as a whole (cf. Plaut and Gonnerman, 2000). In morphologically rich languages, the mappings between form and meaning are straightforward, so that morphological regularities will dominate language processing. Indeed, in the simulation of a morphologically rich language, priming effects extended to semantically opaque items as well (Plaut and Gonnerman, 2000). However, the network could not simulate equivalent priming effects for semantically transparent and opaque items, as we have found in German.

#### Particle separation

German is a verb-second language with an SOV word order (e.g., Haider, 1985) and therefore separates the particle from its stem in finite forms, and places it at the end of the sentence. The particle, which complements the meaning of the complex verb, can thus occur many words after the stem, with an almost infinite amount of material—ranging from complex noun phrases to relative clauses—inserted in between the finite verb and its particle, as in *Der Bub hörte, nachdem er lauthals geschrien und mit den Beinen auf den Boden gestampft hatte, endlich auf/zu* (L: “The boy finally stopped/listened after he had screamed loudly and stamped with his feet”). It is possible that German readers/listeners are used to keeping more than one possible meaning active upon encountering a verb stem.

#### Morphological richness

Interestingly, so far, strong morphological effects have been observed in Hebrew, Arabic, and German, providing evidence that lexical representation in these languages is guided by morphological structure. Indeed, like Semitic languages, German is a “morphologically rich” language among the Indo-European languages. Differences in morphological richness between Germanic languages such as English, Dutch, and German result from typological differences that emerged during language history (Roelcke, 1997). In synthetic languages like Proto-Germanic, morphology dominantly marked the grammatical relations (hence “morphologically rich”). In analytic languages, morphological markedness is reduced (hence “morphologically impoverished”) and is replaced with syntax to mark grammatical relations, such as word order (De Vogelaer, 2007). In this sense, German is “morphologically richer” than other Indo-European languages, since it has kept morphological markers to indicate grammatical functions. For example, particles and prefixes of German complex verbs express the functions of adverbs of place, time, and manner in more analytic languages. Morphological richness—the use of morphology to express syntax—is a language characteristic that makes German more similar to Semitic languages like Hebrew and Arabic than to Indo-European languages.

We therefore stress the importance of cross-language and cross-linguistic evidence in building models of lexical representations. Most psycholinguistic models of lexical representations usually assume that what is true of one language is true of all. However, our results argue for cross-language differences in morphological processing and hence also in lexical representations. We assume that the features of German train native speakers to generalize the morphological form beyond the meaning of a particular whole-word derivation.

Most of the above mentioned pre- and supralexical or connectionist models cannot incorporate the present findings in German, especially not those regarding opaque morphological effects. For example, the convergence of codes view can easily explain the priming effects in the transparent condition due to form-and-meaning overlap (i.e., with both form and meaning similarity with the target). However, we do not see how this approach can explain the occurrence of equally strong effects in the opaque condition that shares form but no/little meaning.

Another conceivable explanation is rooted in the type of associations triggered by primes and targets. Saussure (in Wunderli, 2013) distinguished between syntagmatic and paradigmatic associations. The former result from the different syntactic roles that words take in the same semantic context, such as *verb–noun*, *adjective–noun*, or *preposition–noun* combinations, as in *drink–coffee*, *red–car*, *lay–above*, *fall–down*. By contrast, paradigmatic associations result from the fact that distinct words that share similar meanings occur with the same set of other words. For example, *red* or *blue* co-occur with similar nouns like *flower*, *car*, *skirt*. Therefore, they have a high semantic similarity via these second order associations.

For large text corpora Rapp (2002) showed that first and second order statistical dependencies reflect the distinction between syntagmatic and paradigmatic associations, respectively. Further, a recent computational model of semantic access uses this distinction in terms of a direct association between words (due to Hebbian, syntagmatic, learning), or a large amount of common associates (common, paradigmatic, contextual features) to successfully predict word activation levels (Hofmann et al., 2011; Hofmann and Jacobs, 2014). With respect to the present study, one could argue that opaque and transparent verbs differ in their associative status: opaque verbs may share paradigmatic contexts—not with their base—but with other derivations of their base, while transparent verbs share both a syntagmatic and a paradigmatic associative status with their base. However, future research is necessary to examine whether the syntagmatic/paradigmatic distinction can explain the similar activation of semantically transparent and opaque verbs in our study. For the time being we think that our data are best accommodated in a single-system model that allows for stem access regardless of regularity and semantic transparency. A short description is sketched below.

### Model of lexical representation in German

In the following, we shortly describe the frequency-based model previously suggested by Smolka et al. (for details, see Smolka, 2005; Smolka et al., 2007b, 2009, 2013, 2014). Its main feature is that complex verbs, including regularly and irregularly inflected verbs as well as semantically transparent and opaque derivations are segmented into stem and affixes and are lexically represented via their stems (and affixes).

The model assumes segmentation processes similar to those suggested by models of prelexical processing. We refer to these studies for a detailed description of the nature of early form-to-meaning mappings (cf. Diependaele et al., 2005; Marslen-Wilson et al., 2008; Crepaldi et al., 2010). Importantly, since morphemes are the smallest meaningful units, they emerge as the product of form-to-meaning mappings. In German, letter strings like *zuziehen* (“pull together”) and *erziehen* (“educate”) are segmented into their constituent morphemes regardless of meaning compositionality: *zu-*, *er-*, *zieh*, *-en*. This accounts for our finding of an N250 effect for all prefixed verbs in the morphological conditions of this study, which fits with the interpretation that N250 modulations indicate a “feed-forward prelexical morpho-orthographic segmentation process that operates independently of lexical status and semantic transparency” (cf. Morris et al., 2011, p. 581).

Then the constituents activate their representations at the lexical level, so that both the transparent verb *zuziehen* and the opaque verb *erziehen* are lexically represented via their base {zieh} and affixes {zu}, {er}, and {en}, respectively. Since the target *ziehen* (“pull”) activates the same lexical units {zieh} and {en}, its recognition is facilitated by the prior presentation of a complex verb with the same base. This accounts for our findings that the N400 attenuations induced by morphologically related words are independent of meaning compositionality. This also accounts for our finding that the facilitation in form of N400 modulations by verbs sharing the same base is larger than that by semantically associated verbs holding a different base.

Further, the finding that semantically opaque verbs induce the same amount of facilitation as transparent ones explains why both types of derivation induce an additional P325: Both types of derivations are lexically represented via the stem, just as the base verb is. Hence, the priming effect of the base corresponds to identity priming, which is typically reflected in positivities that precede the N400, such as the P325. For example, the P325 was found in repetition priming studies that used identical prime target pairs like *table-table* (Holcomb and Grainger, 2006) or gender-inflected nouns like *bobo-boba* (cf. Domínguez et al., 2004), see also Table 1.

The finding that semantically opaque verbs induce the same amount of facilitation as transparent ones indicates that the stems were accessed before the meaning of the whole word, which contradicts the assumptions of a supralexical model (e.g., Giraudo and Grainger, 2000; Diependaele et al., 2005). This finding further contradicts the assumptions of distributed-connectionist approaches or the convergence of codes view, according to which semantically transparent words should always yield stronger effects than semantically opaque words (e.g., Rueckl et al., 1997; Plaut and Gonnerman, 2000; Kielar and Joanisse, 2011). These assume that morphological regularities emerge during visual word processing when orthographic codes are mapped onto meaning codes. During this mapping process, the strength of the semantic association is expected to affect the form-to-meaning mappings. Accordingly, semantically transparent derivations should always yield stronger priming effects than semantically opaque ones. However, this was not the case in the present study.

So far, we have explained how complex verbs are segmented (as indicated by the N250) and accessed via their stem regardless of meaning compositionality (as indicated by the P325 and the N400). How is the specific meaning of a complex word derived? If we are aware of the fact that even semantically transparent derivations yield specific idiosyncratic concepts from the meaning of the base and the function of the prefix, we may assume that transparent and opaque meanings are generated in similar manners. The very specific—more or less idiosyncratic—meaning of a complex word is activated by the lexical constituents that represent a word. For example, the stem-affix combination *zieh* (“pull”) and *zu* (“together”) will activate the transparent concept PULL TOGETHER, while the stem-affix combination *zieh* (“bind”) and *er-*^4^ will activate the opaque concept EDUCATE. Note that both concepts differ from the concept PULL of the single constituent.

In our frequency-based model, the specific meanings are selected by mechanisms that rely only on connections between lexical and conceptual units, choosing the most frequently activated concept upon the co-activation of the constituents. It is possible, though, to assume separate whole-word lemmas similar to “*superlemmas*” in idiom processing that are activated by the simultaneous activation of several constituents at the lexical level (e.g., Sprenger et al., 2006; Kuiper et al., 2007; Smolka et al., 2007a; Rabanus et al., 2008). Irrespective of how the specific meaning is activated following lexical access, our findings indicate that the complex verb is lexically accessed and processed via its stem.

In sum, our findings indicate that lexical representation in German refers to the base of a complex verb, regardless of meaning compositionality. This indicates that morphological structure represents an important aspect of language processing in German and must be incorporated in the lexical representation of German words.

## Conflict of interest statement

The authors declare that the research was conducted in the absence of any commercial or financial relationships that could be construed as a potential conflict of interest.

## Footnotes

^1^The N400 effect was originally observed in sentences with semantically non-fitting words. Over the years researchers agreed that the N400 amplitude is also related to additional search processes in long-term memory when the system attempts to find a coherent interpretation for a word, a sentence, a numerical equation, etc. Thus the expression *N400 effect* or *N400 deflection* is usually used to address an increase of a negative-going amplitude in the latency range of 400 ms when stimuli induce semantic activity in memory (see e.g., Kutas and Federmeier, 2011; Rösler, 2011). A reduction of the N400-effect is therefore accepted as a sign of less activity in memory, as it is, for example, the case when a stimulus has been primed.

^2^http://www.neurobs.com/

^3^Since response latencies were collected previously (see Experiment 2a in Smolka et al., 2009), we dispensed with the collection of latencies here so as to assure that the event-related potentials following target presentation were not confounded with brain potentials usually seen for response preparation.

^4^It is under discussion whether German prefixes, which are bound morphemes, have a meaning of their own.
